# Supplementary figures and images for: An experimental assessment of detection dog ability to locate great crested newts (Triturus cristatus) at distance and through soil
Source: PLoS One. 2023 Jun 7;18(6):e0285084. doi: 10.1371/journal.pone.0285084 (PMC10246828; doi:10.1371/journal.pone.0285084)

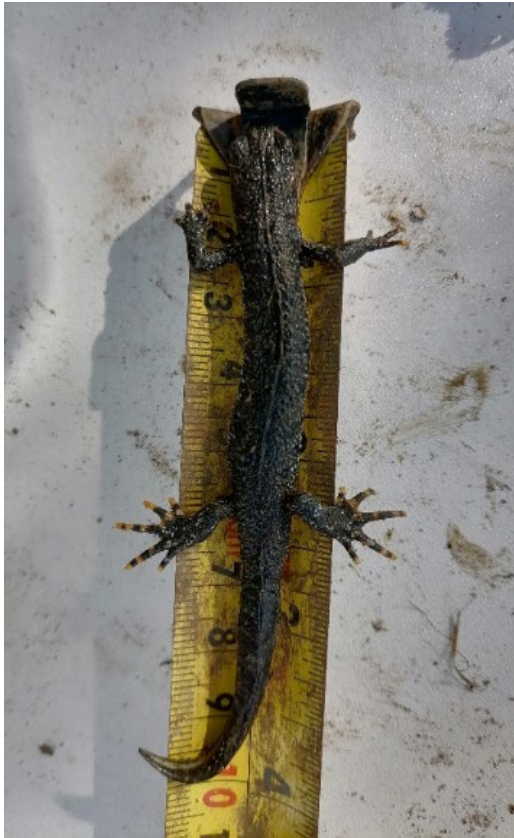

**S1 Fig. *T.cristatus* being measured from nose to cloaca**

Supplement: S1 Fig — (PDF) [file pone.0285084.s001.pdf]
