# Supplementary material for: An experimental assessment of detection dog ability to locate great crested newts (Triturus cristatus) at distance and through soil
Source: PLoS One. 2023 Jun 7;18(6):e0285084. doi: 10.1371/journal.pone.0285084 (PMC10246828; doi:10.1371/journal.pone.0285084)
Supplement: S2 Fig — (PDF) [file pone.0285084.s002.pdf]

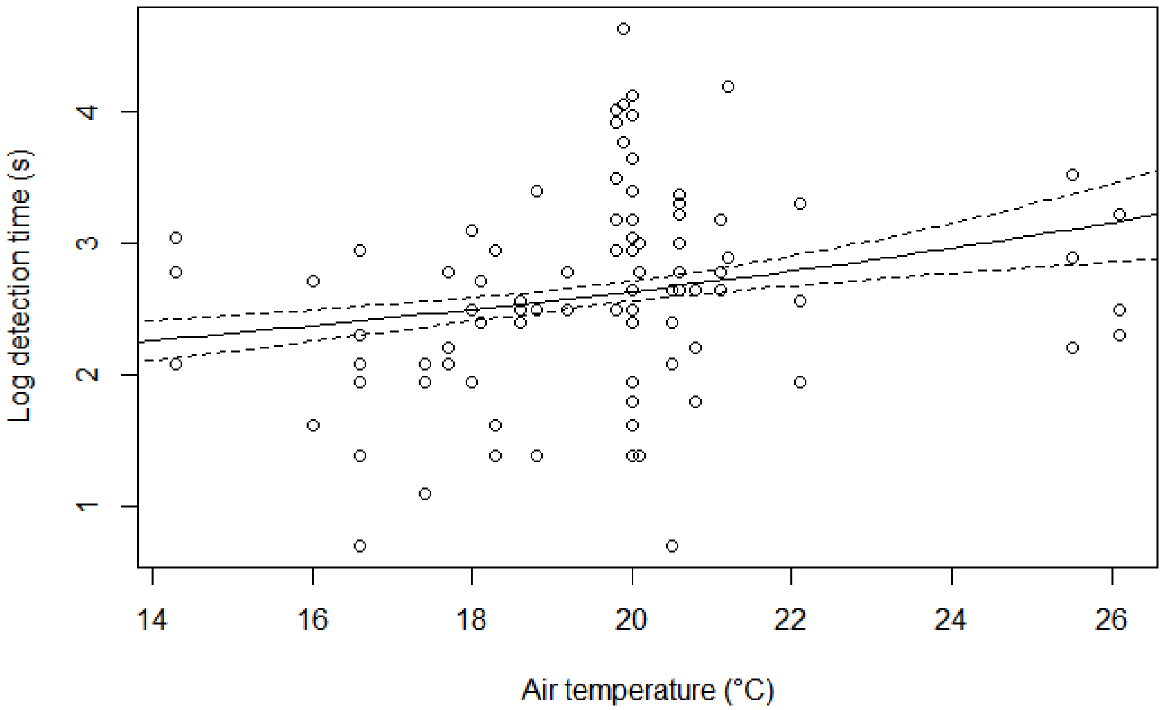

S2 Fig. The relationship between (log) detection time and air temperature with predicted values + SE from a gamma GLM (log effect size = 0.59 ± 0.09, *t* = 6.34, *p* <0.05).
