# Supplementary material for: An experimental assessment of detection dog ability to locate great crested newts (Triturus cristatus) at distance and through soil
Source: PLoS One. 2023 Jun 7;18(6):e0285084. doi: 10.1371/journal.pone.0285084 (PMC10246828; doi:10.1371/journal.pone.0285084)
Supplement: S1 Table — (PDF) [file pone.0285084.s003.pdf]

S1 Table: Results of the weather conditions during the channelled distance perception trials.

Distance (cm)	Time GCN entered tub	Weather conditions	Temp (degree C)	Humidity	Wind Speed (Beaufort scale)	Direction
**25**	14:20	Sunny intervals, dry, 20% cloud	27.8	47%	3	Easterly
**50**	14:20	Sunny intervals, dry, 16% cloud	26.2	47%	3	Easterly
**75**	14:20	Sunny intervals dry, 15% cloud	26.7	43%	3	Easterly
**100**	14:20	Sunny intervals dry, 20% cloud	23.6	57%	3	Easterly
**125**	16:30	Sunny intervals, dry 15% cloud	23.0	44%	3	Easterly
**150**	16:30	Sunny intervals, dry 15% cloud	22.3	55%	3	Easterly
**175**	16:30	Sunny intervals, dry 20% cloud	22.8	56%	5	Easterly
**200**	16:30	Sunny intervals, dry 30% cloud	21.6	60%	5	Easterly
