# Supplementary material for: An experimental assessment of detection dog ability to locate great crested newts (Triturus cristatus) at distance and through soil
Source: PLoS One. 2023 Jun 7;18(6):e0285084. doi: 10.1371/journal.pone.0285084 (PMC10246828; doi:10.1371/journal.pone.0285084)
Supplement: Attachment — Submitted filename: Review.docx [file pone.0285084.s004.docx]

**Introduction**

The introduction is very well written and overall follows a logical flow. My only suggestion would be to start from the ecological point of view (i.e., line 46 – maybe not with a “To”), and once the terrestrial methods for surveying newts are brought up (currently ending line 76), come to the method of detection dogs. This would prevent from this sharp cut that currently exists between paragraph 1 and 2.

line 42: references 6-9: three of the references refer to carcass monitoring. Although this is true, it seems a little less variable to generalize that dogs are generally 4-12 times better than humans. I would suggest that the authors also include papers on other species or their traces.

line 49: please give some references on those “easier” sampling methods

line 51: again the addition of some typical sampling methods and their efficiency would help the readers

line 62: it is also an annex 4 species of the FFH directive

**Methods**

Generally, I would like to congratulate the authors to their very creative testing procedures and apparatuses. This is a great accomplishment and will shed light into the secret of newt detection dogs, if not amphibian detection dogs in general. That said, I have a long list of additional information that is necessary to follow the setups entirely and procedures.

The description of test 1 was quite good while there were several logical flaws in the description of test 2 (see below). I am also missing some information on how habituated the dog was to the apparatuses and the setups, since mistakes often occurred in the first trials.

In contrast to the writing, the concept of test 2 seems well thought-through with 4 conditions and 128 repetitions. This gives room for rather sound statistical analyses. However, in test 1 there were only 2 repetitions for 8 conditions making further analyses almost impossible. Despite the finding is interesting, the concept is also biased towards learning by the dog as the length used per trial was not randomly chosen but increased systematically, limiting the potential to generalize these findings. This should be explained and taken up in the discussion.

Finally, despite test 2 is following a good concept, statistical analyses done afterwards are rather weak. A pairwise comparison of findings that at the same time may (or may not) depend on so many other factors contradicts the test assumptions. I would suggest a linear regression (linear model or an anova) for these analyses, with the detection time as a response and properties of the individuals (individual measurements, sex) and environmental conditions (weather in this case, as long as weather parameters are not correlated, of course) as explanatory variables. After reading the results, I also figured out that the dog did a lot of false-positive indications. I would thus use a binary coding of whether the dog was able to detect the newt at the first attempt (1) or not (0), neglecting whether or not the dog would have found the individual in any following attempt (because the likelihood of detection is different from the first attempt). This binary coding (i.e., detection probability) would then be used as a response in a glm with Binomial error distribution and all the above-mentioned individual and environmental parameters as explanatory variables.

line 87: as experiments were conducted in different year, it may be better to give the year of birth or a reference when the dog was five years old (at the beginning? last test?)

lines 89-90: what is meant by “annual assessments”? How is the dog assessed and what for? Is there any standard protocol the authors could refer to?

line 90: likewise, please define “suitable operating standards”

line 92: this is a good example of how such an assessment can be cited

line 92-93: “Freya is able to distinguish between the target species and all other common amphibians native to the UK” – after having a look at reference 31, it is not clear to me how the authors define “is able to…”. Would the authors consider giving the “score” for Freya? According to ref. 31, it seems that 80% GCN detected and no more than 2 false positives is a “pass”, but 50% or lower GCN detection and 4 or more false positives are a “fail”. That makes it hard for the reader to evaluate the dog performance.

line 100-110: I would like to congratulate the authors to such a very smart setup. This is a great idea. I have a few questions: Would the authors consider also sharing a picture of the bottom of a pipe (description lines 103-106), maybe as an inlay to Fig. 1? Why was the test restricted to two runs per length (which results in very low statistical power)? Was that because 16 individuals were available and no individual should be used more than once? And last, I have some concerns with the “successively increasing the length” as this could result in a learning effect, while on operative searches it is more likely that the dog encounters various lengths randomly. Results may be different if a dog is tested with a successively changing situation (which would rather be the approach of a learning protocol than for a test) in comparison to a randomly changing situation (a short pipe run followed by a long and then a medium pipe run etc.). Last, was the position of the board always the same in respect to the pipe length that exceeded the board? So that for the dog the setup always looked identical?

line 114: please specify “blind” to “double blind”

line 115: please insert: “from the handler” or the observer/tester “to the detection dog” (see below for wording confusion)

line 123: “one additional repeat” – backwards or starting again?

lines 127-129: please specify the parameters taken during the test, e.g. what were the weather conditions and how were they defined, how was temperature / wind speed / humidity measured, what categories existed for the behavior of the dog and handler and how were they defined?

line 130: the “tester” is the same person as the “observer”? Then, I would suggest to use a unique name. If they are not the same, I did not understand the tasks of both so far. Please also verify again which of these persons was placing the newt and then went out of sight of the handler, and whether the third person (if there was any) observed the whole procedure. If so, the test is no longer considered as double-blind but single blind.

line 156: the setup is not visible in Fig. 2a. Would the authors please consider adding a scheme of the setup?

line 161: this may be a funny question, but how is the 68 mm pipe attached to a 75 mm pipe adaptor? It may just be my version of the pdf, but Fig. 2b is a little too blurry to see it

line 164: this sentence gave me a hard time to understand the setup. I suppose that the 1x1 cm holes are supposed to be the mesh size? And the mesh has been rolled to a diameter of 3.75 cm? Again, a drawn scheme would be helpful here.

lines 166-170: this part needs some more details to understand the whole setup. As far as I understood it, 8 pipes per plot existed, one was holding a newt (or more?)? And all other soil types could exist? So two pipes per soil type in each plot? Please specify.

line 173: again this my seem to be a funny question, but if the pipe was 20 cm height and the adaptor was 5 cm height, but the hole was just 22 cm depth, the pipe must protrude 3 cm above surface. Was that on purpose? How does that affect the setup?

lines 175-177: the information about the weighting of the crested newt should go along with line 135 where the newts are mentioned.

lines 178-179: again, please define the parameters properly. Also clarify why you added cloud cover to your set of parameters

line 179: is that soil moisture measurement different from the procedure described above, i.e. has it been repeated during the tests?

line 183: this sounds very negative and pessimistic about the dog. I am sure also the true positives were recorded. ☺ Likewise, the number of misses (false negatives) were likely also recorded (or would be, if there were any). I would appreciate a more complete picture in that sentence.

line 183-184: again I am getting confused with the tasks for tester and observer. Please clarify their tasks and who was present at the plot and when. Also, it should be mentioned that the test was conducted single-blind (despite you described it in the text that the handler did not know the location, readers my search for blindness in your article).

line 186: the dog “undertook a free search” – but actually, Fig. 2a suggests that the dog was working on a long towing leash; please clarify; another upcoming question: how did the dog search? did she deliberately check the 8 tubes? did the handler follow the dog or stood still at the entrance of the plot?

line 186: “dog indicated correctly” – before the handler confirmed with the tester, it is not necessarily correct. Rewrite sentence in more logical order, e.g., dog indicated – handler confirmed with tester – if correct, play session

line 187-190: this sentence seems to be a bit miss-placed and would be more in logical flow after line 191. I suggest to change this.

line 188: If I understood the setup correctly, there was always one empty plot among the four plots. Was the handler able to stop the search before the 180 sec and state that she believes that nothing is here? please clarify.

line 191: and also the number of attempts as well as the time until the dog would find the newt was noted, I guess? please specify

line 194: I think I am not yet familiar with what the authors determine as “pipe” and “tube”. While line 192 suggests that new tubes were used during each trial, line 194 states that all pipes were moved to new positions. Please clarify this issue and specify the terms used.

line 197: how were the 30 newts distributed among the 128 trials (even though some were without newts)? was the selection completely random or pseudo-random (i.e. allowing for a similar number of trials per newt)? On another note, there is again a wording issue with “trial”, which the authors sometimes use for all four plots together and sometimes for a single plot (at least this is how I understand the 128 trials). Maybe the authors could think of a more appropriate term, or specify (as done sometimes but not throughout the manuscript) as 4-plot and single-plot trials

statistical analyses: the statistical part should follow the same order as the field methods part. It would thus be better to start with statistics of test 1; if no statistics for test 1 were done, please at least describe some potential measures for this test; unfortunately, more repetitions would have been helpful for a more comprehensive understanding

line 200: what “data” are the authors talking about? To understand this part, a general context on what the authors aim to analyse would help. For example, differences among detection rates or detection times regarding the soil type etc. Were the detection times checked for normality? Or what else?

line 201: I would suggest to delete the part: “and box plots were used to visualize the data” as this is obvious information

line 203: medians of what?

line 204: differences of what?

line 205: aren’t vents/ no vents and sol/clay the four treatments? What is the difference to the KW-Test?

line 206: how do the authors define detection rates? which parameters belong to the abiotic and biotic parameters? The last part can be added to my question above about a more detailed parameter definition

**Results**

Results are well written but hard to generalize in their current state. Please consider a more sophisticated statistical approach as the one suggested in the Methods part. Also, original data are missing and the authors should consider uploading them to a repository when publishing the article.

Table 1: I forgot to ask: how long was the dog trained on the setup before? How used was she to search on it? Again, due to the successive increasing of the pipe, a learning effect cannot be excluded here.

line 228: soil surface or somewhere within the soil?

line 227-232: despite this information is very interesting, it does not tell much without the corresponding outcome. A summary with the outcome data would help, be it detection probability or time to detection

line 237: more than 20% indication in blank plots seems quite high and would be difficult in real deployments when only retreats can be found but not the newts themselves. Any ideas why this happened? Could residual odor play a role? Again, also the search strategy would be interesting to know, i.e. whether the dog systematically checked the tubes (by herself or with a handler following).

line 240-241: this statement must be underlined with statistics, otherwise it is too blurry. See my suggestions about a potential statistical analysis in the Method Section

line 242: as for test 1, I am wondering how familiar the dog was to this setup.

Figure 3: please add pairwise significant differences to the plot

lines 244-251: directions (effect sizes) are entirely missing. If things are significantly different, which one was faster/slower?

lines 251-253: again, please underline the statements with statistical results

**Discussion**

General: The discussion is very well written and follows a red line. Unfortunately, discussions of own results are rather short (despite including very nice parts, see below). If the authors would consider a full analysis on detection at first attempt and detection time depending on all parameters measured (as suggested above), results would be much more comprehensive and could directly be compared to more findings from others. In line with that, the authors claimed that they did not detect any differences between sexes or with size, which contradicts recent findings from a different detection dog on GCN (Grimm-Seyfarth 2022). Having a look at the whole models (sometimes parameter influences can only be detected when controlling for other, maybe more important parameters, such as potentially the soil type in this case) and comparing results would be extremely interesting.

A conclusion is missing.

line 265: and limited to a single amphibian species

line 281: careful, the authors did not evaluate what is commonly referred to as detection distance. I typical detection distance would be when an individual / trace is placed and the dog would wind its head and change direction at xx m distance and then move straight to the target. The authors however used a lineup with different pipe lengths, were the smell would not be distributed through the environment but channeled in a pipe, likely coming close to a chimney effect (Osterkamp 2000). The amount of smell through such a pipe is much higher than would be on the surface. Nevertheless I do understand the applicability of these findings when newts are hidden in the ground. Your findings tell us that the dog would find newts in 2 m deep mammal burrows (if they would be straight etc.), which is a very important finding. However, I would strongly recommend that the authors use a proper term for it to not confuse it with typical detection distances.

line 288: careful, reference 39 is talking about detection distances (measured accurately with GPS and in field-like setting), while the authors tested the distance channeled through a pipe. this is not really comparable and should be specified

line 290: reference 41 evaluated the distance from a defined transect line that a scat can be detected, thus also not really evaluating detection distance (which would properly be done like in ref. 39); please specify

line 291: it would be helpful to not just know the temperature range exhibited during the test, but also whether hot temperatures occurred at the beginning or end, as temperature may influence the channeling effect of the scent

line 292: Grimm-Seyfarth 2022 investigated the effect of temperature (among others) on detection probability for T. cristatus, which apparently was also dependent on the habitat; and my suggestion would be to include temperature analyses on test 2 (see my suggestion in methods part)

lines 298-308: these are important information, thanks for including them! I would appreciate to have some more information in the method section as requested above, as this may clarify some of my points risen above

line 311: the studies by Matthew are indeed quite comparable to some points and I would suggest to draw some more direct comparisons regarding her and your results

312-326: this is a very nice part of the discussion! I would encourage the authors to include more of that. Comparisons among studies, even if different approaches have been used, and their findings are extremely helpful in this field.

**References**

I did not check all the references, but this came to me:

line 415: there seems to be a mistake with the title, see <https://www.sciencedirect.com/science/article/pii/S0006320703002684?via%3Dihub>

**References given**

Grimm-Seyfarth, A. 2022. Environmental and training factors affect canine detection probabilities for terrestrial newt surveys. Journal of Veterinary Behavior 57: 6-15.

Osterkamp, T. 2020. Detector dogs and scent movement. How weather, terrain, and vegetation influence search strategies. CRC Press, Boca Raton, USA.
